# Fire Eater's Pneumonia: One of the Rare Differential Diagnoses of Pulmonary Mass Images

**Published:** 2011-03-30

**Authors:** F. Şahin, P. Yıldız

**Affiliations:** 1 Pulmonologist, Department of Pulmonology, Yedikule Chest Diseases and Thoracic Surgery Research and Training Hospital, Istanbul, Turkey; 2 Associate Professor of Pulmonolgy, Department of Chest Disease, Yedikule Chest Diseases and Thoracic Surgery Research and Training Hospital, Istanbul, Turkey

Dear Editor,

Accidental aspiration of liquid hydrocarbon mixture by animators who blow flame may lead to severe lipoid pneumonia.[1][2][3] This situation that occurs due to accidental aspiration of liquid hydrocarbon mixture (gas oil, gas oil-cognac mix) during animation shows is called “Fire Eater’s Pneumonia” in the literature, which has been presented in few cases.[1][2][4][5] Different consequences may occur through the high and low viscosity products.[5][6] In cases reported in the literature, the main clinical symptoms are cough, dyspnea, chest pain and fever; the subjects are generally referred to the emergency room with severe dyspnea.[7][8][9][10] Hereby, we introduce two cases of fire eater’s pneumonia. 

**Fig. 1:**
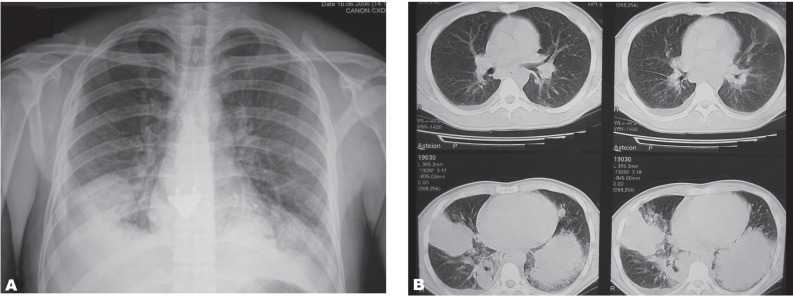
A 29-year-old male patient with fire eater’s pneumonia. (A. Partial opacities eliminate the heart and diaphragmatic contours in the bilateral lower zones in his chest radiography. B. Minimal pleural liquid is seen on the right side with dense and solid parenchymal masses in the bilateral lower lobes, density increases in the form of ground glass together with pulmonary nodules in thoracic CT.)

The first case was a 29-year-old male animator who aspirated liquid gas during his flame-blowing show in a circus. He was admitted to the emergency room with productive cough, fever and severe dyspnea. He had taken lamb oil in his mouth 18 hours ago and blew this mix to a stick in order to conduct a flame-blowing show. Immediately after the show, the cough had started. In the chest radiography, partial opacities eliminating the heart and diaphragmatic contours in bilateral lower zones were determined (Fig. 1A). Thoracic CT showed right sinus blunting due to pleural effusion along with right side opacities and lesser opacities on the left side. In spiral CT of the thorax, minimal pleural liquid was seen on the right side with dense and solid parenchymal masses in the bilateral lower lobes, density increases in the form of ground glass. In addition, pulmonary nodules were found (Fig. 1B). The patient improved clinically after a ten-day treatment; however, regular chest x rays showed partial improvement during this period until the first month radiography that showed no pathology except for slight elevation of the right diaphragm.

**Fig. 2:**
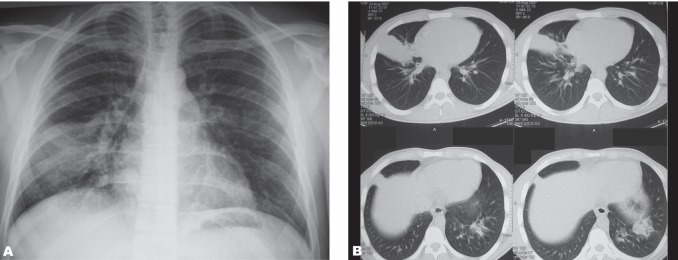
A 22-year-old male patient with fire eater’s pneumonia.( A. A relatively homogeneous density was determined without eliminating the heart and diaphragm contours in his chest radiography. B. Consolidation areas covering the lateral segment of the right middle lobe together with small nodules in the medial segment of the middle lobe and lateral basal segments of the lower lobe. Bronchopneumonic consolidation areas in the left lower lobe are detected in thoracic CT.)

The second case was a 22-year-old male animator similar to the patient mentioned above admitted with productive bloody cough, fever and severe dyspnea. The cough had started 24 hours after the show, fever had developed 2 days after the show and the bloody expectoration had started 15 days later. In chest radiography, a relatively homogeneous density was determined without eliminating the heart and diaphragm contours (Fig. 2A). At the end of the second day, his fever disappeared and, in the chest x-ray the lesions had regressed slightly. In the thoracic CT, a consolidation area completely covering the lateral segment of the right middle lobe was seen together with small nodules in the medial segment of the middle lobe and lateral basal segments of the lower lobe. In addition, bronchopneumonic consolidation areas in the left lower lobe accompanied by peribronchovascular thickenings were seen (Fig. 2B). In the first week, the patient’s clinical and laboratory findings improved. Regular chest x-rays were taken and at the end of the first month the chest x-ray had improved completely. The high viscosity products including paraphine had caused a pseudotumoral exogenous lipoid pneumonia. On the other hand, it has been claimed that low viscosity products may cause acute infection.[6] Fire eater’s pneumonia generally develops by aspiration of low viscosity hydrocarbons such as kerosene, gasoline and turpentine.[7][8] 

They are seen in the form of radiological one sided or bilateral local or diffused perihilar and especially lower lobe consolidation, nodules with good contours, pneumatocells, atelactatic areas, tumor like lesions and rarely pleural effusion.[11][12][13][14] Broncho-pleural fistula and spontaneous pyopneumothorax development were reported in one patient.[13] CT is an important imaging in the evaluation of the characteristics and prevalence of different lesions, their location and characteristics of the pleuropulmonary complications, and especially assessment of the cases who do not respond to treatment well.[13][15] 

In case 1, the radiological improvement was determined at the end of the first month. No pathology was seen except for slight elevation of the right diaphragm in chest x-ray taken one month later. In the thoracic CT taken after 6 months, no radiological abnormality was seen except for the fibrotic scar adjacent to the right diaphragm interpreted as pleural effusion sequela. In the second case, radiological improvement was also seen one month later. When we look at the observation results of the cases reported in the literature, similarly we see that Burkhardt et al reported no pathology apart from the small scar lesions 2 months after initiation of the symptoms. Other case presentations also did not report any pathology excluding the limited sequels.[1][4][9][10]

Consequently, fire eater’s pneumonia should be considered in the radiological differential diagnosis of pulmonary mass-like lesions. The diagnosis is very easy with careful evaluation of the anamnesis and clinical characteristics. Radiological improvement happens slower and later than the clinical improvement. Therefore, patients should be followed with radiological assessments.
